# SurfR: Riding the wave of RNA-seq data with a comprehensive bioconductor package to identify surface protein-coding genes

**DOI:** 10.1093/bioadv/vbae201

**Published:** 2024-12-14

**Authors:** Aurora Maurizio, Anna Sofia Tascini, Marco J Morelli

**Affiliations:** Center for Omics Sciences, IRCCS San Raffaele Scientific Institute, Milan 20132, Italy; Center for Omics Sciences, IRCCS San Raffaele Scientific Institute, Milan 20132, Italy; Universita‘Vita-Salute San Raffaele, Milan 20132, Italy; Center for Omics Sciences, IRCCS San Raffaele Scientific Institute, Milan 20132, Italy; Universita‘Vita-Salute San Raffaele, Milan 20132, Italy

## Abstract

**Motivation:**

Proteins at the cell surface connect signaling networks and largely determine a cell’s capacity to communicate and interact with its environment. In particular, variations in transcriptomic profiles are often observed between healthy and diseased cells, leading to distinct sets of cell-surface proteins. For these reasons, cell-surface proteins may act as biomarkers for the detection of cells of interest in tissues or body fluids, are often the target of pharmaceutical agents, and hold significant promise in the clinical practice for diagnosis, prognosis, treatment development, and evaluation of therapy response. Therefore, implementing robust methods to identify condition-specific cell-surface proteins is of pivotal importance to advance biomedical research.

**Results:**

We developed SurfR, an R/Bioconductor package providing a streamlined end-to-end workflow for computationally identifying surface protein-coding genes from expression data. Our user-friendly, comprehensive workflow performs systematic expression data retrieval from public databases, differential gene expression across conditions, integration of datasets, enrichment analysis, identification of targetable proteins on a condition of interest, and data visualization

**Availability and implementation:**

SurfR is released under GNU-GPL-v3.0 License. Source code, documentation, examples, and tutorials are available through Bioconductor (http://www.bioconductor.org/packages/SurfR). RMD notebooks with the use cases code described in the manuscript can be found on GitHub (https://github.com/auroramaurizio/SurfR_UseCases).

## 1 Introduction

Surface Proteins (SPs) are proteins located on or within the cell plasma membrane that orchestrate cell-to-cell communication and cell interactions with the extracellular environment. SPs have many functions, including nutrient and ion transport, intercellular signaling, enzymatic reactions, and immune surveillance. Due to their specific localization in a restricted spatial, temporal, and functional space, SPs can serve as informative biomarkers for early disease detection, diagnosis, and prognosis and represent promising targets for immune therapy development. Notably, over 60% of approved drugs for human diseases target SPs (Knox *et al.* 2024). However, even though the genes coding for SPs, or Surface Protein-Coding Genes (SPCGs), represent ∼20% of all genes (da Cunha *et al.* 2009), only a fraction of SPs are currently used as targets for FDA-approved drugs or are in clinical trials, thus offering significant margins of improvement for new discoveries and advancements.

Techniques for studying and characterizing SPs include flow cytometry, immunohistochemistry (IHC), immunofluorescence (IF), Western blot (WB), and mass spectrometry (MS). Each of these methods has specific limitations. For example, WB, IHC, and IF rely on high-specificity antibodies and are generally applicable to a limited number of *a priori* known SP targets. MS, while powerful for identifying and quantifying proteins, can be complex and expensive, and as a consequence, publicly available datasets are scarcer (Chandramouli and Qian 2009). Therefore, the identification and prioritization of targetable SPs and their characterization across physiological and pathological tissues remain challenging, while studying SP transcripts is much more straightforward. Indeed, advancements in technology and the reduction in costs have made RNA-sequencing of human samples easy and affordable, with many datasets already available in public repositories.

Leveraging this wealth of data, several computational methods for SPCGs detection from RNA-seq data have been proposed. However, even these bioinformatic approaches face limitations, such as code readability, documentation, and pipeline portability, compatibility, and scalability. Specifically, many works outline intriguing approaches for prioritizing cell-type-specific markers, but authors fail to share the accompanying code, hindering the reproducibility of their results (Town *et al.* 2016, Pais *et al.* 2019, Xu *et al.* 2020, Zhang *et al.* 2020a, Syafruddin *et al.* 2021). Other authors do share the source code used to perform the analysis, yet these scripts are frequently incomplete or inadequately documented, making it difficult and time-consuming to reproduce the original results and, more importantly, to apply the methods to new datasets (Mercatelli *et al.* 2021). Comprehensive R and Python packages for the study of SPCGs exist only for single-cell RNA-seq data (Zhou *et al.* 2020, Javaid and Frost 2023), which often suffers from sparsity and dropout events, leading to incomplete capture of gene expression at the single-cell level, potentially missing SPGGs expressed at low levels. Software (Hong *et al.* 2018) and online graphical user interface (GUI) platforms (Bausch-Fluck *et al.* 2018, Waas *et al.* 2020) are also available but require rigid data formatting and have scalability issues when processing vast amounts of data.

Therefore, the systematic identification and prioritization of new SPCG candidates performed on large collections of sequencing samples still lack rigorous and documented bioinformatic procedures. To address this gap, we have developed SurfR, an R package that can detect SPCGs from bulk RNA-seq data. SurfR is now available in the Bioconductor repository, from Bioc Version 3.19. The package is compatible with Linux, MacOS, and Windows, ensuring accessibility for a wide range of users. SurfR is an open-source, automated, Bioconductor-peer-reviewed, documented, and streamlined pipeline to predict and prioritize SPCGs from transcriptomics data. With SurfR, data stored in public repositories, such as the Gene Expression Omnibus (GEO) (Edgar *et al.* 2002) and The Cancer Genome Atlas (TCGA) (Weinstein *et al.* 2013), can be automatically downloaded and analyzed to unveil specific SPCG expression patterns across organs and tissues in both physiological and pathological conditions, exploiting available data collections and large-scale multidimensional experiments. Furthermore, SurfR facilitates comparative studies and result integration via meta-analysis, thus producing robust and statistically sound results, as well as taking into account the biological and technical variabilities within studies, along with additional study-specific effects.

## 2 Methods

**Figure 1.**
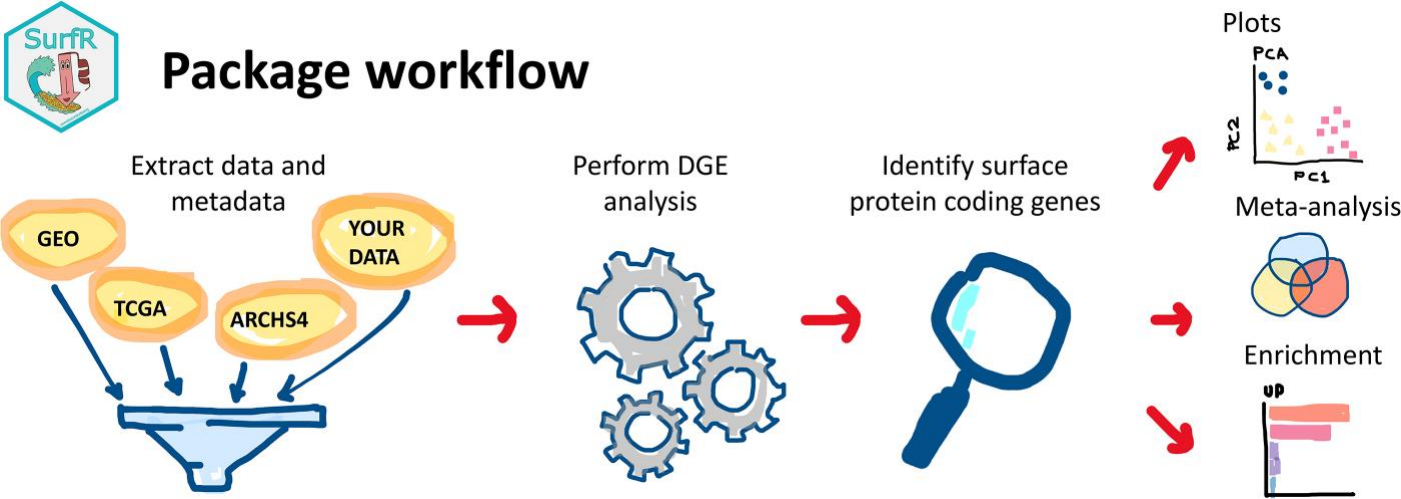
SurfR package workflow.

SurfR is a tool for comprehensively analyzing bulk transcriptomics data to quickly and easily identify SPCGs. The full SurfR analysis workflow is straightforward and consists of a series of steps, illustrated in Fig. 1. Users are guided through the entire process, from retrieving raw data to performing Differential Gene Expression analysis (DGE) and identifying SPCGs via a comprehensive tutorial depicted in the package vignette (the performances of SurfR in running the vignette are available in the online supplementary material).

### 2.1 Identifying SPCGs

The core function of SurfR returns a list of SPCGs, starting from a list of genes of interest. SurfR classifies SPCGs using SURFY (Bausch-Fluck *et al.* 2018), a resource for the prediction of protein sub-cellular localization that can be interrogated using an online GUI (wlab.ethz.ch/surfaceome) allowing users to access an underlying database of Machine Learning (ML)-predicted and experimentally validated SPs. With the web server, the SURFY database can be queried at the single-gene level or by manually uploading lists of genes. While SURFY’s web interface is user-friendly for small-scale queries, it becomes less practical for large-scale analyses due to the need for manual input and one-at-a-time queries. Currently, the output is intended for visual inspection only, as there is no option for export. Therefore, users need to highlight, copy, and paste the output data if they desire to save it and post-process it. SurfR streamlines the process of interrogation of the database through the use of an *ad-hoc* R function called Gene2Sprotein, which provides scalability by enabling a high-throughput and automated analysis, which is particularly useful for large datasets and comprehensive studies. Users can either input to the function a list of genes or automatically derive the list from RNA-seq data, as well as post-process the output within the same framework to produce descriptive plots and enrichment analyses in a scriptable and customizable environment.

### 2.2 Collecting public data

SurfR makes obtaining public data extremely smooth: within SurfR, it is possible to automatically retrieve data from public repositories such as GEO (functions GEOmetadata and DownloadArchS4) and TCGA (function TCGA_download). GEO queries are based on the ArchS4 pipeline (Lachmann *et al.* 2018), which allows the download of uniformly processed data across multiple studies programmatically. Conversely, the TCGA repository is interrogated through TCGAbiolinks (Mounir *et al.* 2019), an R/Bioconductor package designed to automatize TCGA data retrieval, analysis, and visualization.

### 2.3 Performing the DGE analysis

Starting from the RNA-seq raw count matrix, within SurfR, it is possible to perform a DGE analysis across two conditions (typically pathological versus physiological) to identify the genes overexpressed in the condition of interest. This operation is performed with the DESeq2 package (Love *et al.* 2014), which is wrapped in the SurfR function DGE. An ideal biomarker gene, which could also serve as a potential therapeutic target, must have high expression in the pathological samples and almost null expression in controls. To detect the best candidates, the output data.frame of the DGE function contains information on the average expression of each gene in the two groups (Test-T and Control-C), measured in counts per million mapped reads (the Mean_CPM_T and Mean_CPM_C columns of the DGE function output) along with log2FoldChange and adjusted p-value (*p_adj_*).

### 2.4 Integrating multiple datasets via meta-analysis

SurfR offers the opportunity to increase the sample size of a cohort by integrating multiple datasets, therefore enhancing the power to detect differentially expressed genes (DEGs) of interest. This can be achieved by performing a meta-analysis through the metaRNASeq function, which considers inter-study variability that may arise from technical differences among experiments (e.g. sample preparation, library protocols, batch effects), as well as additional biological variability. This function is based on the metaRNASeq Bioconductor package (Rau *et al.* 2014) and implements two different techniques for *P*-value combination (inverse normal and Fisher methods) to increase the detection power for the identification of DEGs. Within SurfR, we employ Venn diagrams as a visual tool to show overlapping genes across datasets. Nevertheless, when comparing multiple RNA-seq datasets, quantitative integration with meta-analysis is a more thorough approach, especially when the user needs rigorous statistical validation, or to account for study heterogeneity. This becomes crucial when the goal is to identify consistent signals across studies with varying sample sizes and design.

### 2.5 Enhancing biological understanding

Gene ontologies (GO) and pathway annotations can also be performed within SurfR to gain further insights about SPCG candidates, using the function Enrichment. In parallel, the list of interest can be characterized at the gene level with cross-database identifiers and descriptions (EntrezID, Uniprot, KEGG, etc.), taking advantage of the 35 gene-set libraries present in the Enrichr database, using the SurfR built-in function Annotate_SPID. Both functions are based on the enrichR cran package (Kuleshov *et al.* 2016).

### 2.6 Plotting the results

Finally, SurfR features several functions to visualize the distribution of SPCG related data and enrichment results, and to compare outputs from different studies, including bar plots (functions Splot and Enrichment_barplot), Venn diagrams (function Svenn), and principal components analysis (PCA) plots (function plotPCA) to help users visualize and interpret their findings.

## 3 Use-case

In order to show the potential of SurfR, we downloaded two public bulk-RNA-seq cholangiocarcinoma datasets, from GEO (GSE107943) and TCGA (TCGA.CHOL) repositories, respectively. Data were recursively downloaded with SurfR functions as specified in the online supplementary material. The GSE107943 dataset includes 57 RNA-seq samples: 30 cholangiocarcinomas and 27 controls consisting of tumor-adjacent tissue. The TCGA.CHOL dataset consists of 35 cholangiocarcinoma samples and 9 paracancerous samples, representing tissues that were adjacent to malignant cells but did not exhibit cancerous characteristics themselves, used as controls.

**Figure 2.**
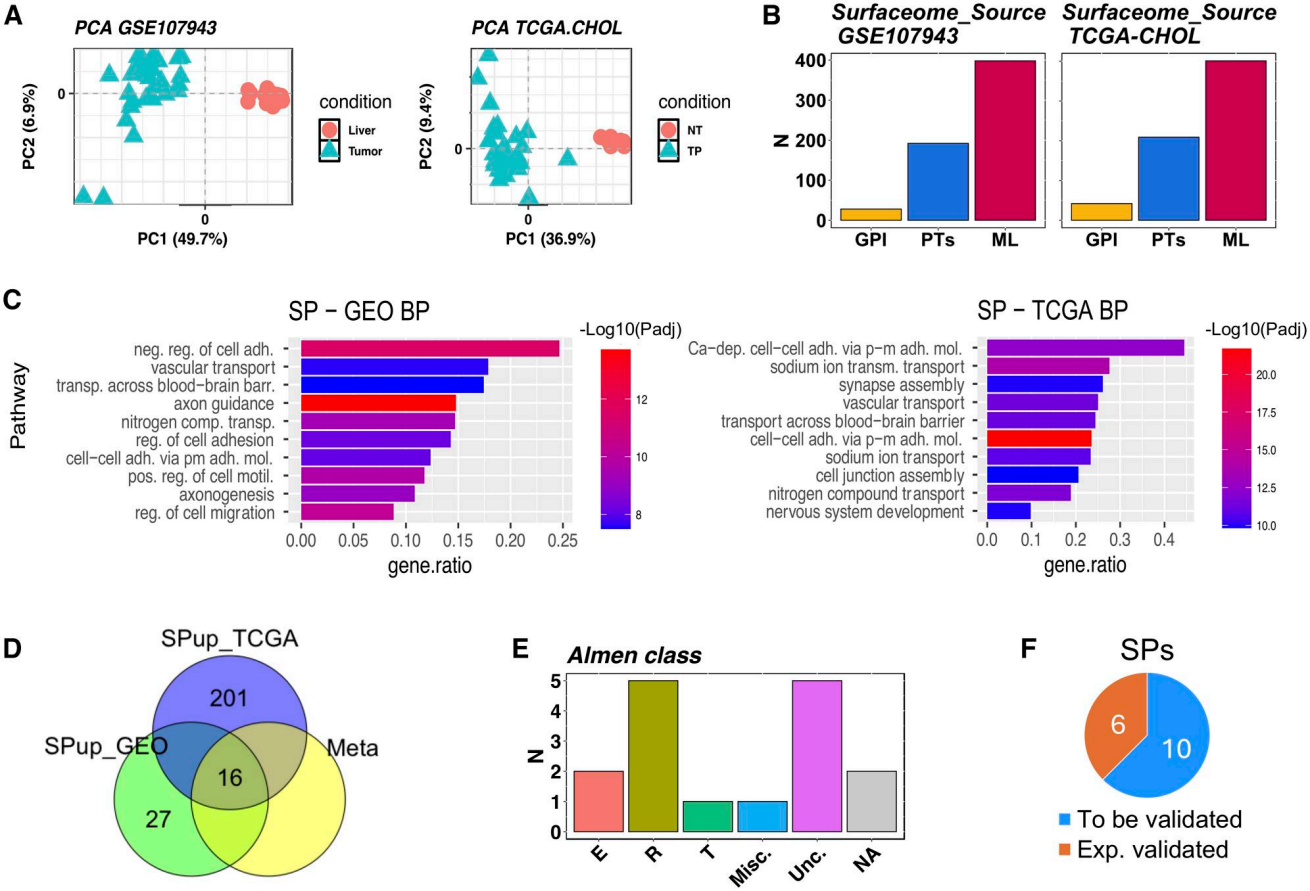
(A) PCA plots generated by SurfR::plotPCA. Left panel: GSE107943 samples (Tumor = tumor tissue, Liver = paracancerous tissue). Right panel: TCGA-CHOL samples (TP = tumor tissue, NT = paracancerous tissue); (B) Barplot, produced by SurfR::SPlot function, showing the Surfaceome.Label.Source (GPI = SPs annotated in Uniprot, PTs = SPs belonging to the training set used for the ML model of SURFY, ML = SP predicted by the ML algorithm of SURFY) distribution of predicted SPCGs in GEO (left panel) and TCGA (right panel) databases; (C) Barplots generated with the SurfR function Enrichment barplot representing the ten most significant up-regulated pathways of the GO Biological Pathways database, within a tumor/normal comparison subsetting SPs, for two cholangiocarcinoma datasets [GSE107943 (left) and TCGA-CHOL (right)]; (D) Venn diagram generated using SurfR::SVenn, illustrating the common up-regulated surface proteins in tumor versus normal comparisons across the GSE107943 and TCGA-CHOL datasets, and their combined results through meta-analysis, filtered based on low expression in the control group (Mean CPM < 0.5); (E) Barplot, produced by SurfR::SPlot, showing the Almen class distribution of the 16 surface protein-coding genes in the intersection of GSE107943 and TCGA-CHOL datasets [E = enzymes, R= receptors, T = transporters, Misc = miscellaneus, Unc = Unclassified (reported as not classified by Almen *et al.* (2009)), NA = proteins not included in the Almen *et al.*’s classification]. (F) Current status of the 16 upregulated SPs shared by the two datasets. More details on the six experimentally validated proteins are provided in Supplementary Table S4.

To gain insights into the datasets and evaluate the presence of batch effects within each cohort, we performed a PCA with the function plotPCA. Both datasets showed a clear separation of tumor and tumor-adjacent normal samples within the first principal component (Fig. 2A). Using the SurfR::DGE function, we performed a DGE analysis of tumor samples versus control samples for each dataset and considered genes significantly modulated if padj < 0.05. The sign of the log2FoldChange field was used to differentiate up-regulated from down-regulated genes.

With SURFY, interrogated via SurfR::Gene2Sprotein function, we identified SPCGs within the list of filtered, upregulated genes. Overall, reanalyzing the GSE107943 dataset with SurfR, we identified 5972 genes up-regulated in the tumor, 618 of which encoded for a SP, either experimentally validated (220) or predicted by ML (398) (Fig. 2B, left panel and Supplementary Table S1). In the TCGA-CHOL dataset, we identified 6265 up-regulated genes in the tumor, 652 which encoded for SP, either experimentally validated (251) or predicted by ML (401) (Fig. 2B, right panel and Supplementary Table S2). Four hundred seven SPCGs were in common across the two datasets, as shown in Fig. S1 of online supplementary material. The enrichment analysis of modulated genes from both datasets accurately reflects the expected biological characteristics of tumor and control samples, as described in Supplementary Fig. S2. SPCG candidates found in the GEO and TCGA datasets were compared using a Venn diagram produced with Svenn to increase the detection accuracy (Supplementary Fig. S5C).

To evaluate whether our dataset reliably represents the collection of cell SPs, we performed a GO analysis from genes encoding for SPs significantly up-regulated in the tumor samples, and genes not classified as SPs (nSPs), considering those with |log2FoldChange| > 1. As shown in Fig. 2C and in Supplementary Fig. S4, we found that pathways related to the roles of SPs, such as cell–cell adhesion, ion transport, cell junctions, integral component of plasma membrane and receptor activity were significantly enriched in our datasets of computationally predicted SPCGs. This is true for SPCGs associated to validated SPs annotated in UniProt and for SPCGs associated to SPs predicted by ML. On the other hand, nSPs genes enriched pathways related to DNA replication, extracellular matrix, and fiber organization (Supplementary Figs S3 and S4). This aligns with our expectations, demonstrating that SurfR operates effectively in identifying SPCGs associated with SPs.

Good markers are unambiguously expressed by a specific cell-type in a condition of interest and almost not expressed in the background. To prioritize strong biomarker candidates, we applied a filter to retain only DEGs with a very low average expression in the control groups, setting Mean CPM C < 0.5. With this procedure, we obtained 43 candidate SPCGs in the GEO dataset, and 217 in the TCGA dataset. In Fig. 2D, we show the intersection of the filtered SPs in the two databases, composed of 16 SPCGs, which represent 37% of the filtered SPCG genes in the GEO dataset (Fig. 2D). The GEO dataset, the one containing the lowest number of SPCGs, set therefore the upper bound in the number of potential candidates resulting from the combination of the two datasets. This intersection ratio could be due to several factors, such as differences in sample types and experimental conditions.

Utilizing the SurfR function Splot, we visualized as a barplot both the surfaceome label source and the classes defined in Almen *et al.* (2009) (Fig. 2E), reported in the data.frame produced by the Gene2Sprotein function to gain further insights on the reliability of the prediction and the role of the identified SPCGs (enzymes, receptors, transporters, etc.).

To enhance the reliability of the results and increase the statistical power of detection, we performed a meta-analysis of the two RNA-seq experiments using the SurfR::metaRNAseq function, as described in the online supplementary material. TCGA and GEO results combined by meta-analysis resulted in 16 candidate genes with a specific expression in tumor tissue compared to normal adjacent tissues; most of these genes were found to be receptors (as shown in Fig. 2E). The number of SPCG candidates identified from the integration of multiple datasets is significantly influenced by the stringency of the filtering criteria, as well as by the biological and technical differences across studies, regardless of the integration strategy. Interestingly, the 16 SPCGs emerging via meta-analysis and the 16 SPCGs obtained intersecting the two datasets analyzed separately coincided, suggesting robustness and reliability in the identification of candidates across different datasets and analytical approaches (Supplementary Fig. S5D). This overlap strengthens the confidence in these 16 SPCGs as potential biomarkers or therapeutic targets for cholangiocarcinoma, as their differential expression is consistently observed across multiple datasets. The agreement between the meta-analysis and the intersecting approach validates the selected candidate genes and reinforces their relevance in the context of the studied condition.

As an additional confirmation of the validity of our approach, we report that 6 out of 16 of these genes encode for SPs, which were experimentally validated with complementary techniques such as WB, qRT-PCR, IHC, and immunoblotting in the context of cholangiocarcinoma or other cancer types (Fig. 2F). In particular, these candidate genes include MUC4 (Shibahara *et al.* 2004), LYPD1 (Chen *et al.* 2020), NOX4 (Weyemi *et al.* 2012), Neurotrimin (Eun *et al.* 2023), I17RD (Asukai *et al.* 2015), and GIPR (Waser *et al.* 2012), as detailed in Supplementary Table S4. The remaining SPCGs represent a sound starting point for future investigations and targeted validation.

Running the entire analysis for this use case took <10 min on a macOS Monterey laptop (x86_64, R version 4.3.1), and no parallelization was employed. The most time-consuming step was downloading data from public databases, which accounted for a total of 1.5 min.

## 4 Discussion

We have developed SurfR, an R/Bioconductor package, to provide a streamlined end-to-end workflow for *in-silico* identification of SPCGs from RNA-seq data. Our user-friendly, comprehensive workflow also features the possibility to perform systematic retrieval of expression data from public databases, differential gene expression analysis across conditions, integration of datasets, enrichment analysis, and data visualization for an intuitive interpretation of the results. We illustrated the performance of SurfR by analyzing two cholangiocarcinoma datasets downloaded from GEO and TCGA, respectively, and we identified several SPCGs expressed in cholangiocarcinoma, including experimentally validated SPs. Based on this evidence, we believe that SurfR can become a key asset for the identification of reliable surface markers. Our package can be applied across different contexts and human pathologies beyond cancer, where specific subsets of cells exhibit distinct SP expression profiles, such as autoimmune diseases, infectious diseases, and neurodegenerative disorders. Within these conditions, SPs mediate processes such as inflammation, immune activation, and angiogenesis, which are also central to cardiovascular diseases. For example, cardiomyocytes differ functionally across regions of the heart, and some diseases, like regional heart failure, affect specific cell populations only. When treatments are not targeted to these populations, they can cause unintended side effects (e.g. a treatment for atrial fibrillation may induce ventricular arrhythmias). Mapping the cardiomyocyte surfaceome across different heart regions could help prioritize drug targets, including cell-type-specific, maturation-stage-specific, and region-restricted SPs.

We provide here an example of SP identification in the context of neuroscience in Supplementary Fig. S6 based on the work of Weiss *et al.* (2016).

In these scenarios, often the amount of data collected in an institution is the limiting factor to unravel the complexity of a problem. SurfR may help in this regard by increasing the numerosity of samples through data retrieval from public databases, while also addressing the challenge of data integration via meta-analysis.

It must be noted that gene expression values do not necessarily translate into a quantity of protein, and this is particularly true for SPCGs for which the mRNA–protein correlation is less accurate than for cytoplasmic proteins (Greenbaum *et al.* 2003). The high protein turnover and low half-lives observed in SPCGs reflect the cellular ability to react quickly to extracellular stimuli. Since RNA-seq only measures the transcriptome, the correlation between RNAs and proteins may potentially be low because of a series of factors, like stability and lifetime differences of the two types of molecules, post-transcriptional/post-translational modifications, the difference between the total cellular pool of a protein and its actual abundance at the cell surface, protein turn-over, and proteolytic processing. Among the technical issues influencing the discordance between RNA and protein levels, there are the context-dependent off-target binding of the antibodies used to isolate the cell type of interest in a complex tissue, and the biases in the mapping of the RNA-seq reads over the genome. Future package developments could explore integrating proteomics datasets, such as MS data, into SurfR to further refine SPCG predictions.

Working with mRNA has on the other hand many advantages, especially when the aim is to explore the transcriptome without a predefined list of target SPs of interest, including the costs and scalability of RNA-seq data, and the ability to detect low-abundance proteins as surfaceome genes with low expression that are below the current detection limit of mass-spectrometry-based methods. An mRNA-based approach would therefore be very useful in identifying the genes that can be otherwise missed in a proteomics-based approach (Edfors *et al.* 2016). Additionally, the mRNA analysis allows for a fast and relatively cheap exploration of dynamic changes in gene expression patterns, providing insights into the regulation of SPs under various conditions. Finally, a distinctive aspect of using an RNA-seq-based approach is that it can be applied to meta-analysis of the large amount of published RNA-seq data, which greatly outnumbers proteomics datasets.

Our package also has some limits: it has been developed to streamline the analysis of quantitative transcriptomics data where often a mixture of cell populations is present. When samples lack purity or purity information, identifying specific cell types expressing SPCGs can be challenging. For instance, an SPCG overexpressed in a tumor sample may originate from infiltrating cells, not tumor cells. This issue complicates RNA and protein level correlations in heterogeneous samples without prior cell sorting. In these cases, we suggest using matched scRNA-seq datasets or consulting public scRNA-seq resources like Tabula Sapiens (The Tabula Sapiens Consortium 2022) to identify cell types expressing genes of interest and refine candidate gene lists for further validation. Deconvolution approaches (Newman *et al.* 2015, Tsoucas *et al.* 2019, Menden *et al.* 2020) could also be employed to assess cell-type proportion within each condition and estimate cell-type-specific gene expression. Global changes in gene expression between two conditions can result from alterations in the tissue’s cellular composition, changes in gene expression within specific cell populations, or a combination of both. Deconvolving the cell types present in a sample from changes in gene expression is critical in systems characterized by cellular proliferation (e.g. cancer), cell death (e.g. neurodegenerative diseases), and in inflammatory conditions or tissue remodeling processes.

When combining public databases, users must be aware of potential batch effects, which can arise from differences in experimental conditions across studies. SurfR tackles this challenge by downloading raw count data from GEO processed uniformly using the ArchS4 bioinformatics pipeline, ensuring consistent data handling across different studies. Additionally, the SurfR function plotPCA provides a visual tool to detect potential batch effects within datasets. This feature allows users to estimate the magnitude of batch effects and thus to evaluate if and which batch removal techniques are necessary. Specifically, when batch effects across datasets are severe, ComBat-seq (Zhang *et al.* 2020b) may be particularly effective for addressing substantial systematic differences; on the other hand, when the batch variability is limited, it may suffice to incorporate it into the DESeq2 design formula.

For studies combining data from different sources, particularly those with varying sample sizes or quality, we recommend performing a meta-analysis with SurfR function metaRNASeq. This approach is the most robust when merging datasets with such differences, as it allows for the integration of results while accounting for study-specific variability.

The purpose of this manuscript is to present a comprehensive and easy-to-use toolkit for researchers who are interested in predicting SPCGs. SurfR is an open-source, end-to-end workflow, which can streamline tasks that previously needed to be tediously assembled and manually curated. Being a modular toolkit, its application is not limited to the identification of SPCGs, but it also enables quick comparative studies and guides the user in each step of the analysis of both in-house and public bulk-RNA-seq data.

## Supplementary Material

vbae201_Supplementary_Data

## Data Availability

The data underlying this article are available in the GEO repository at https://www.ncbi.nlm.nih.gov/geo/query/acc.cgi?acc=GSE107943 and can be accessed with the accession code *GSE107943* and in TCGA, TCHA-CHOL datasets. SurfR is released under GNU-GPL-v3.0 License. Source code, documentation, examples, and tutorials are available through Bioconductor (http://www.bioconductor.org/packages/SurfR). RMD notebooks with the use cases code described in the manuscript can be found on GitHub (https://github.com/auroramaurizio/SurfR_UseCases).
