# Supplementary material for: SurfR: Riding the wave of RNA-seq data with a comprehensive bioconductor package to identify surface protein-coding genes
Source: Bioinform Adv. 2024 Dec 14;5(1):vbae201. doi: 10.1093/bioadv/vbae201 (PMC11671034; doi:10.1093/bioadv/vbae201)
Supplement: vbae201_Supplementary_Data [file vbae201_supplementary_data.zip › SurfR_SI.docx]

**Public data download**

We used the functions GEOmetadata and DownloadArchS4, respectively, to retrieve the sample metadata and the raw count table of the GSE107943 dataset.

For the TCHA-CHOL dataset, we relied on the TCGA_download function.

**Enrichment analysis of modulated genes - tumor vs normal**

We performed a functional enrichment analysis to improve the biological interpretation of up-regulated or down-regulated DEGs (defined considering p_adj < 0.05 and |log2FoldChange|> 1), using the SurfR built-in function SurfR::Enrichment on the database GO_Biological_Process_2021.

Gene count ratios and enrichment scores were graphically represented as bar plots using SurfR::Enrichment_barplot function.

Up-regulated genes in the tumor show an up-regulation of known tumor pathways, such as cell replication, migration, mitosis, DNA-repair, and extracellular matrix remodeling. These processes are essential for tumor growth, metastasis and evasion of immune surveillance, and are common in most cancer types. Conversely, pathways up-regulated in the controls (tumor-adjacent, healthy tissue of the liver of the same patients) were mostly associated with sterol, fatty acids, and cholesterol metabolism and were more indicative of the homeostasis of the specific tissue at hand in this case, the liver (as shown in Fig S2).

**Meta-analysis**

In order to combine the GSE107943 and TCGA-CHOL studies to increase the statistical power of the detection, we performed a meta-analysis of the two RNA-seq experiments using the SurfR::metaRNAseq function.

The function automatically produces and saves as PDF documents the histograms of raw p-values for each of the individual differential analyses performed using the independent filtering from DESeq2 package (Fig. S5 A,B). The results of the meta-analysis are summarized in a data.frame highlighting the statistical significance for the common genes to all methods (Fisher test e invnorm), using the built-in SurfR function combine_fisher_invnorm. Additional details can be found in the metaRNASeq Bioconductor package vignette (<https://rdrr.io/rforge/metaRNASeq/f/inst/doc/metaRNASeq.pdf>). Genes displaying opposite, and therefore contradictory, log2FoldChange in the two studies were identified (column signFC= 0) and removed from the list of DEGs via meta-analysis.

**Prioritizing and characterizing SPCGs**

These biomarker candidates were further prioritized and characterized using the SurfR Annotate_SPID function, revealing that many of these genes had previously been annotated in the context of carcinomas. Notably, we found hits for these SPCGs in the DSigDB, LINCS_L1000_Chem_Pert_Consensus_Sigs, and IDG_Drug_Targets_2022 databases (see Supplementary Table S3).

**SurfR Beyond Cancer – neuroscience use case**

Code available on GitHub:

<https://github.com/auroramaurizio/SurfR_UseCases/tree/main/SurfR_Neuro>

To show the potential of SurfR in a neuroscience-related context, we reanalyzed the GEO GSE90711 dataset, where the authors performed both transcriptomics and proteomics analyses to identify peripheral nerve injury human Schwann cell SP markers.

Data were recursively downloaded with the SurfR functions GEOmetadata and DownloadArchS4 to retrieve the sample metadata and the raw count table, respectively. The GSE90711 dataset included 27 RNA-Seq samples. We focused on two subsets of data: (i) one composed of peripheral nerve-associated, highly purified, and isolated cell cultures, which include 5 Schwann cell samples and 3 fibroblast samples; (ii) the second consisting of peripheral nerve fascicles pulled out from freshly explanted nerve pieces, consisting of 3 injured (nerve fascicles 1-3 hours after excision) and 3 CTRL (ex vivo pre-regeneration period of 8 days) samples.

To gain insights into the datasets and evaluate the presence of batch effects within each cohort, we performed a PCA with the function plotPCA. Both datasets showed a clear separation across conditions on the first principal component (see Fig.S6A).

Using the SurfR::DGE function, we performed a DGE analysis for each dataset, and considered genes significantly modulated if padj < 0.05. The sign of the log2FoldChange field was used to differentiate up-regulated from down-regulated genes. With SURFY, interrogated via SurfR::Gene2Sprotein function, we identified SPCGs within the lists of filtered, upregulated genes in (i) Schwann cells and (ii) injured fascicles.

Overall, re-analyzing the peripheral nerve-associated primary cell cultures set (i) with SurfR, we identified 2845 genes up-regulated in the Schwann cells, 495 of which encoded for an SP, experimentally validated (161) of ML-predicted (334).

In the peripheral nerve fascicles dataset (ii), we identified 1373 up-regulated genes in the injured samples, 251 of which encoded for SPs, either experimentally validated (102) or predicted by ML (149).

Then, we looked for SPCGs common across the two datasets, as in the Weiss et al. manuscript (Fig. S6B), to delve into the close relationship between Schwann cells and regenerating nerves. SurfR::SPlot function was then used to represent the Almen class distribution of the 83 common SPs (47 ML-predicted, and 36 experimentally validated), Fig. S6C. Among these were detected several established SP markers expressed by Schwann cells during peripheral nerve injury to promote repair, involved in cell adhesion, cell-cell interactions, migration, clearance from debris, and antigen presentation, including NGFR, GFRA1, NRCAM, MERTK, HLA-DOA, MDGA1, FLRT3, TENM3, and ITGB3. Importantly, these nine genes have been validated by immunofluorescence and high-resolution protein profiling (hr-MS) by the authors of the paper associated with the re-analyzed dataset.

The enrichment analysis of modulated genes with | log2FoldChange | > 1 from both datasets performed with SurfR:: Enrichment and graphically represented with SurfR::Enrichment_barplot functions accurately reflect the expected biological characteristics of Schwann cells (enriched pathways included axonogenesis, cell-cell adhesion, myelination, axon-guidance, synapse assembly, cell-junction) and injured peripheral nerve (enriched pathways reflected regenerative processes and included extracellular matrix, mitotic splindle, and collagen fibril organization), as described in Fig. S6D.

To evaluate whether our dataset reliably represents the collection of cell SPs, we performed a GO analysis from genes encoding for SPs common to both datasets. As shown Fig. S6E, we found that pathways related to the roles of surface proteins, such as lytic vacuole membrane, integral component of plasma membrane, and cell-cell adhesion, were significantly enriched in our datasets of computationally predicted SPCGs.

The distinction between the cell surface markers being unambiguously expressed versus overexpressed in a specific cell-type depends on the context of the study. Unambiguous expression is critical when specificity and precision are the goals (e.g., for targeted therapy, disease diagnosis, or cell isolation).

SPCG overexpression, on the other hand, may be sufficient when SPs are used in combination with other markers in functional or exploratory studies, or when comparing states or conditions, as in this use case. The authors of the manuscript associated with this study identified genes that were significantly overexpressed in the cell type and condition of interest, pointing to key biological processes, even if the genes were not uniquely expressed.

To prioritize critical biomarker candidates, even though expressed at low levels, we nevertheless applied a filter to retain only DEGs with a very low average expression in the control groups, setting Mean CPM C < 0.5. With this procedure, we obtained 43 candidate SPCGs in the cell-culture dataset, and 217 in the fascicle dataset. In Fig. S6F, we show the intersection of the filtered SPs in the two datasets, composed of 3 SPs. Considering that Schwann cells represent only a fraction of the cell types present in the nerve fascicle and the stringent threshold, it is not surprising that only a small number of SPCGs were identified. In particular, as shown in Fig. S6G, these candidates include two receptors (HTR1E, MCHR1), among which HTR1E is known to be expressed in Schwann cells in healthy and regenerating nerves (<https://doi.org/10.1016/S0006-8993(96)01411-4>), although it had not yet been validated by the authors of the GSE90711 dataset at the time of publication, and HLA-G, which is consistent with Schwann cells' antigen-presenting and local immune response-modulating function in post-traumatic peripheral nerves (<https://www.nature.com/articles/s41598-017-12744-2>).

**Performance of SurfR**

**On the package vignette**

R version 4.4.1 (2024-06-14) -- "Race for Your Life"

Platform: x86_64-pc-linux-gnu

SurfR.Rcheck/SurfR-Ex.timings.

| **name** | **user** | **system** | **elapsed** |
| --- | --- | --- | --- |
| **Annotate_SPID** | 50.190 | 5.043 | 71.430 |
| **DGE** | 3.222 | 0.176 | 3.399 |
| **DownloadArchS4** | 0.925 | 0.099 | 24.976 |
| **Enrichment** | 0.259 | 0.034 | 3.805 |
| **Enrichment_barplot** | 0.285 | 0.048 | 1.801 |
| **GEOmetadata** | 0.322 | 0.028 | 0.351 |
| **Gene2SProtein** | 11.115 | 1.347 | 12.463 |
| **SVenn** | 0.004 | 0.015 | 0.020 |
| **Splot** | 6.517 | 1.228 | 7.745 |
| **TCGA_download** | 2.920 | 0.215 | 12.711 |
| **combine_fisher_invnorm** | 0.01 | 0.00 | 0.01 |
| **enrichr_download** | 0.096 | 0.019 | 13.255 |
| **metaRNAseq** | 0.004 | 0.000 | 0.005 |
| **plotPCA** | 0.430 | 0.044 | 0.474 |

**On unit tests**

SurfR unit tests

SurfR.Rcheck/tests/testthat.Rout

| **name** | **user** | **system** | **elapsed** |
| --- | --- | --- | --- |
| **Unit.tests proc.time()** | 31.070 | 2.177 | 33.237 |
