# Supplementary material for: SurfR: Riding the wave of RNA-seq data with a comprehensive bioconductor package to identify surface protein-coding genes
Source: Bioinform Adv. 2024 Dec 14;5(1):vbae201. doi: 10.1093/bioadv/vbae201 (PMC11671034; doi:10.1093/bioadv/vbae201)
Supplement: vbae201_Supplementary_Data [file vbae201_supplementary_data.zip › Figure_SurfR_SI.pptx]

## Slide 1
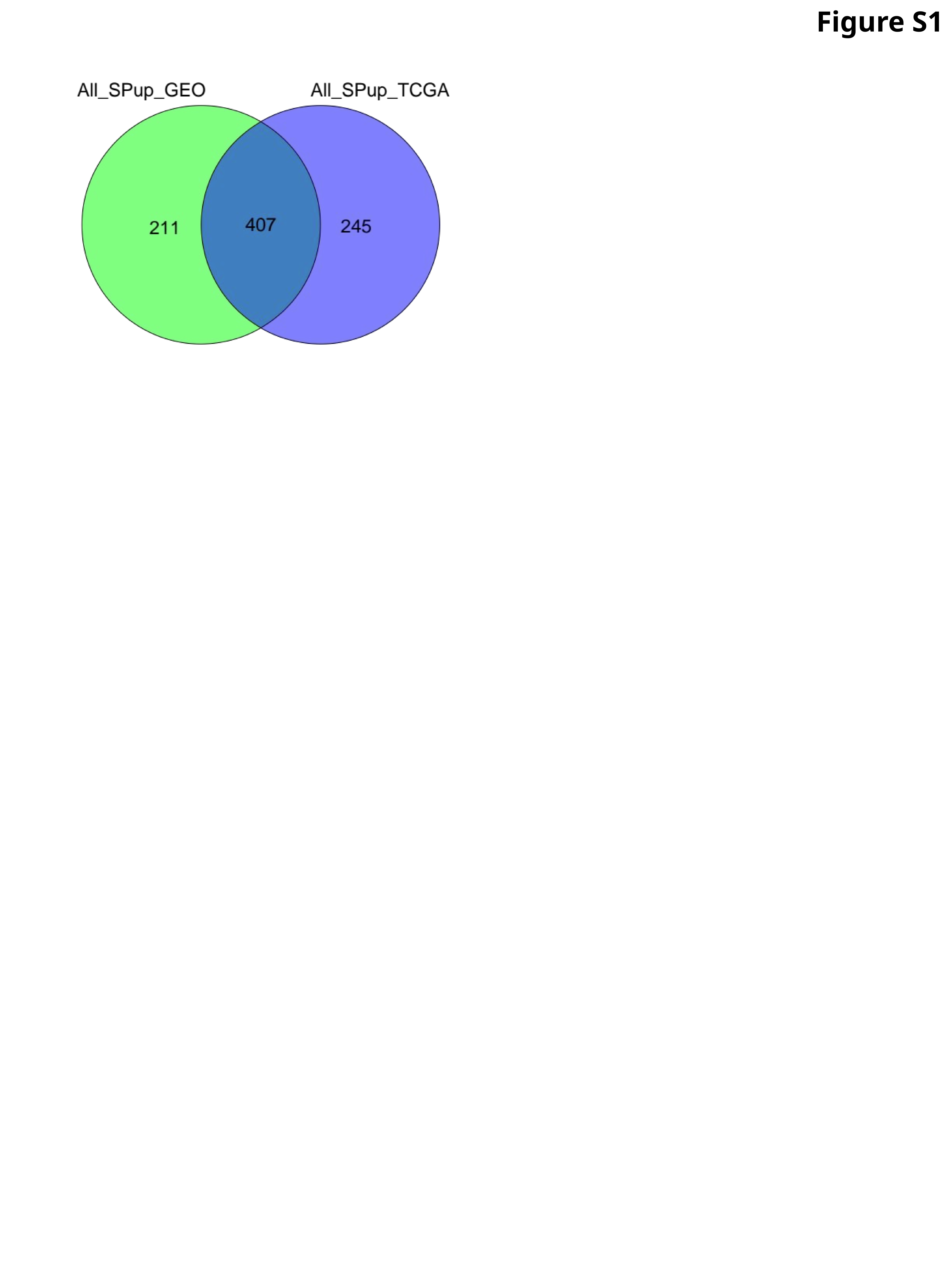

Figure S1

## Slide 2
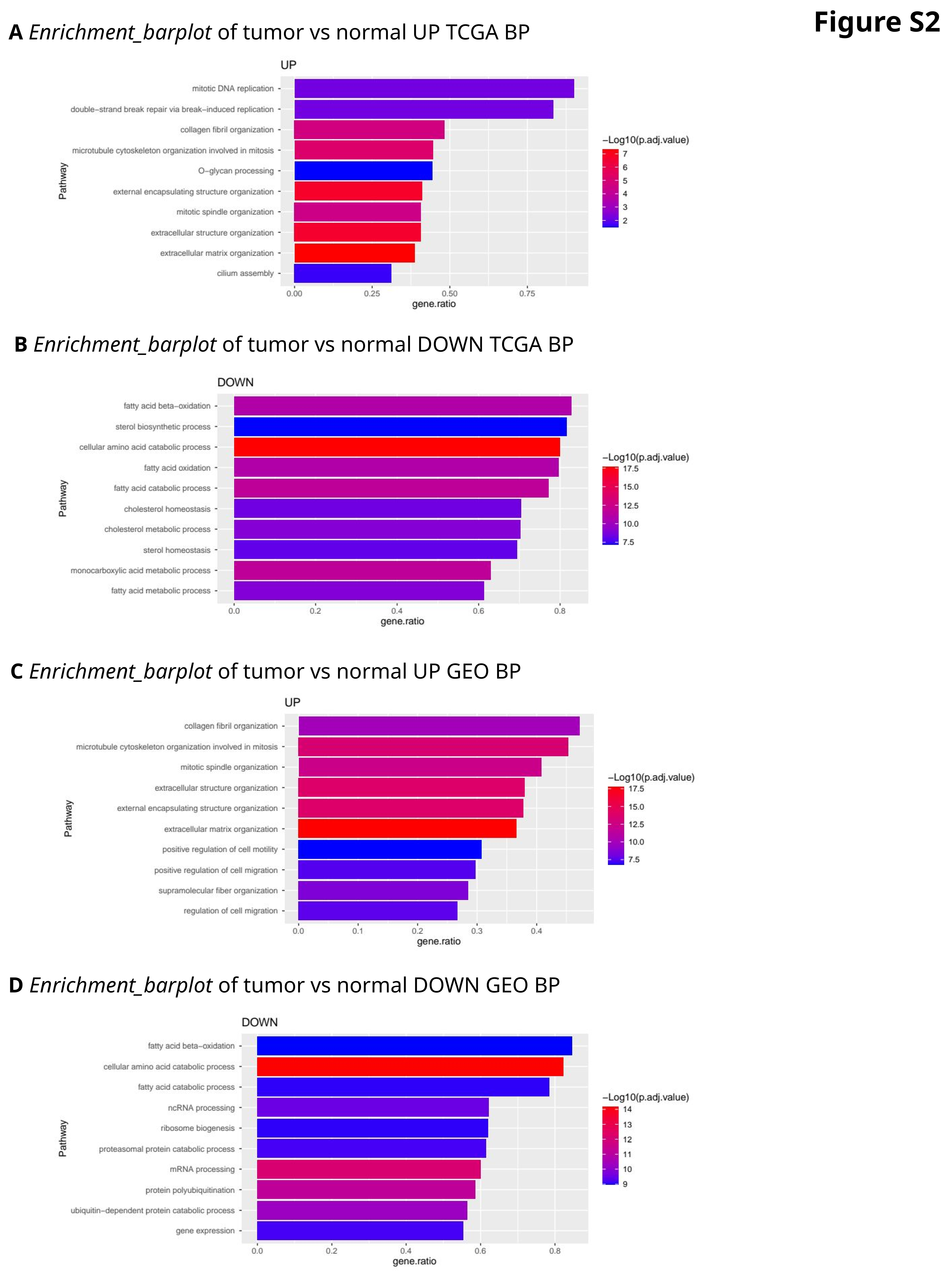

Figure S2
A Enrichment_barplot of tumor vs normal UP TCGA BP
B Enrichment_barplot of tumor vs normal DOWN TCGA BP
C Enrichment_barplot of tumor vs normal UP GEO BP
D Enrichment_barplot of tumor vs normal DOWN GEO BP

## Slide 3
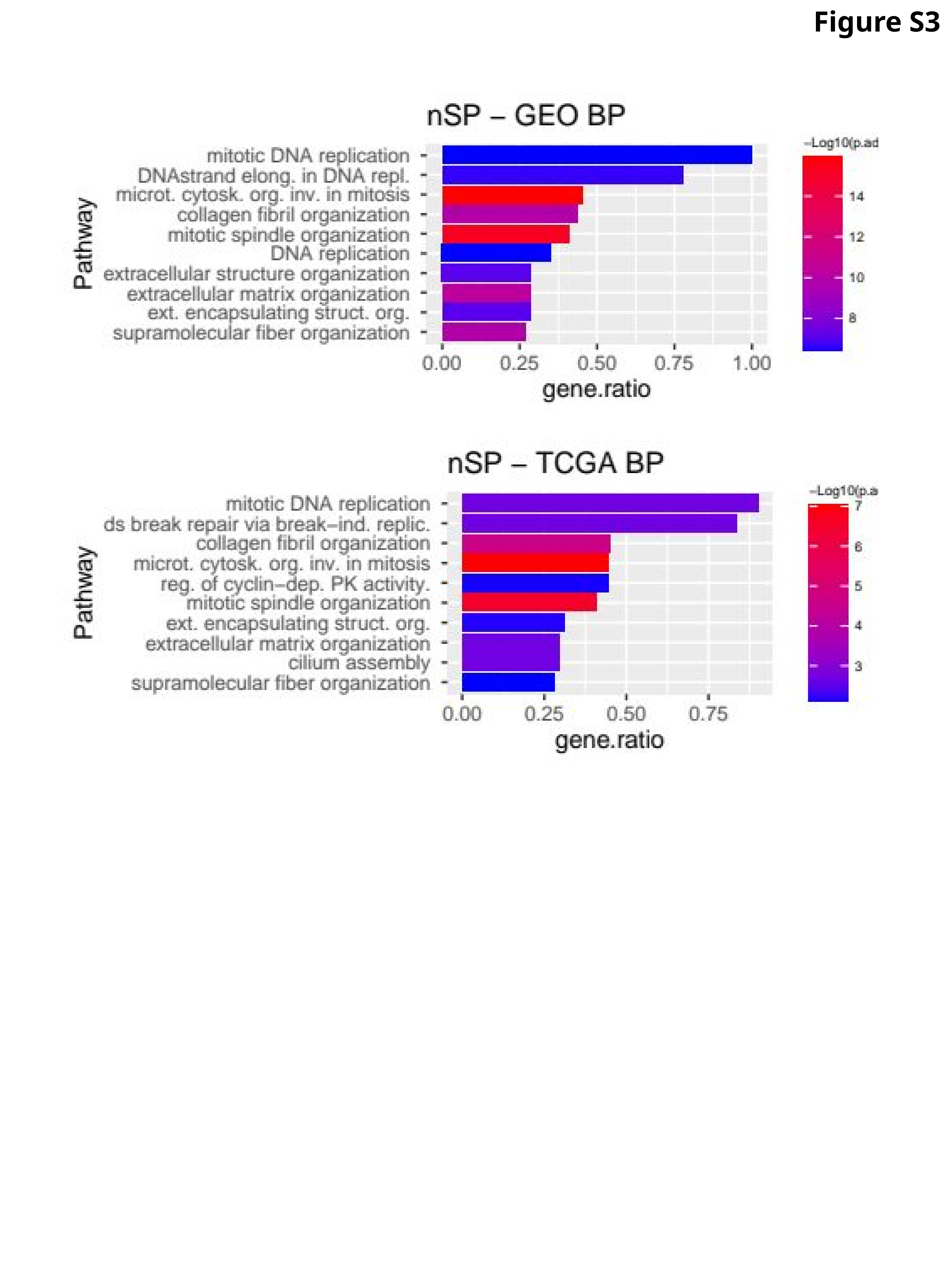

Figure S3

## Slide 4
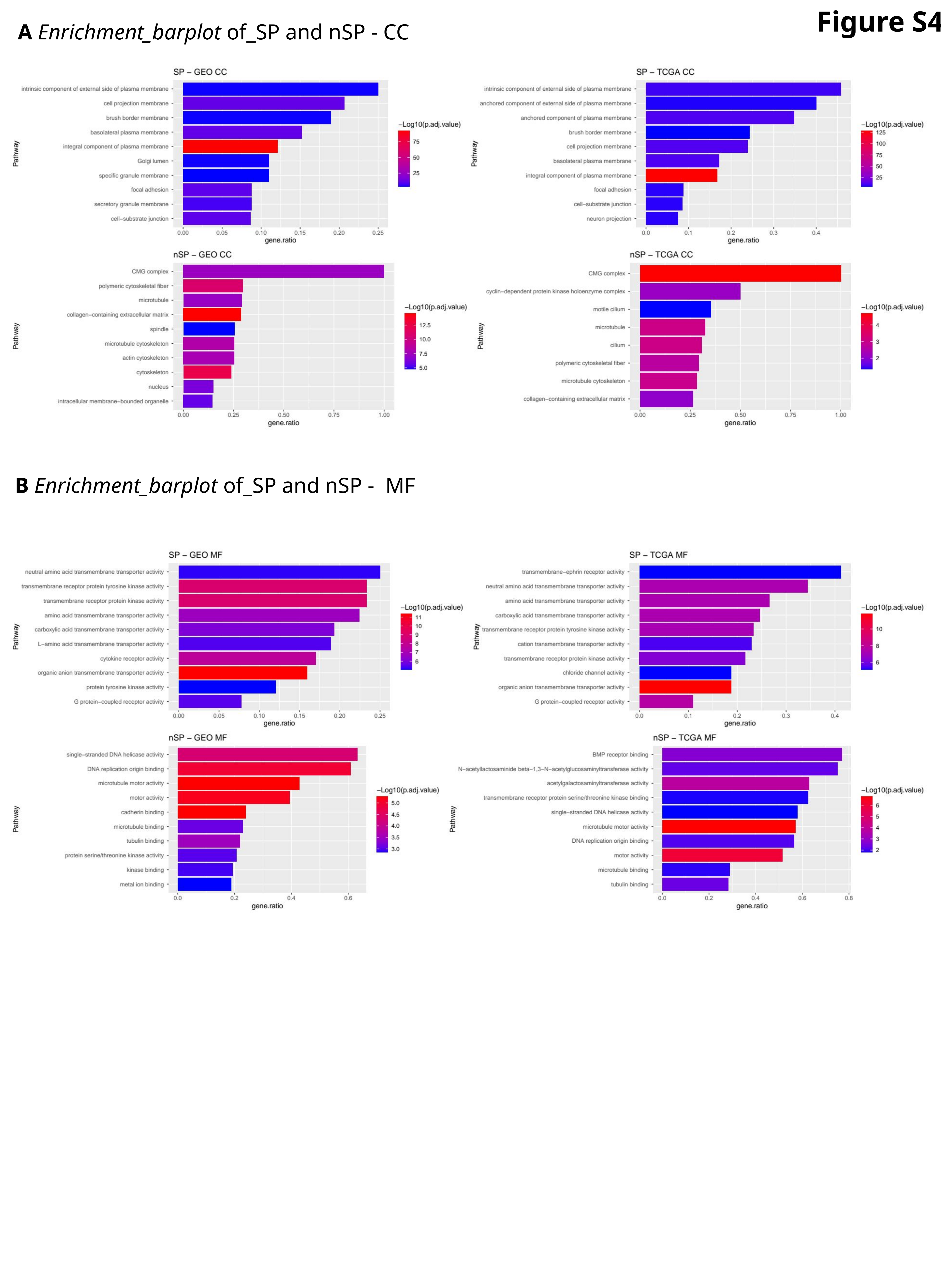

Figure S4
A Enrichment_barplot of_SP and nSP - CC
B Enrichment_barplot of_SP and nSP - MF

## Slide 5
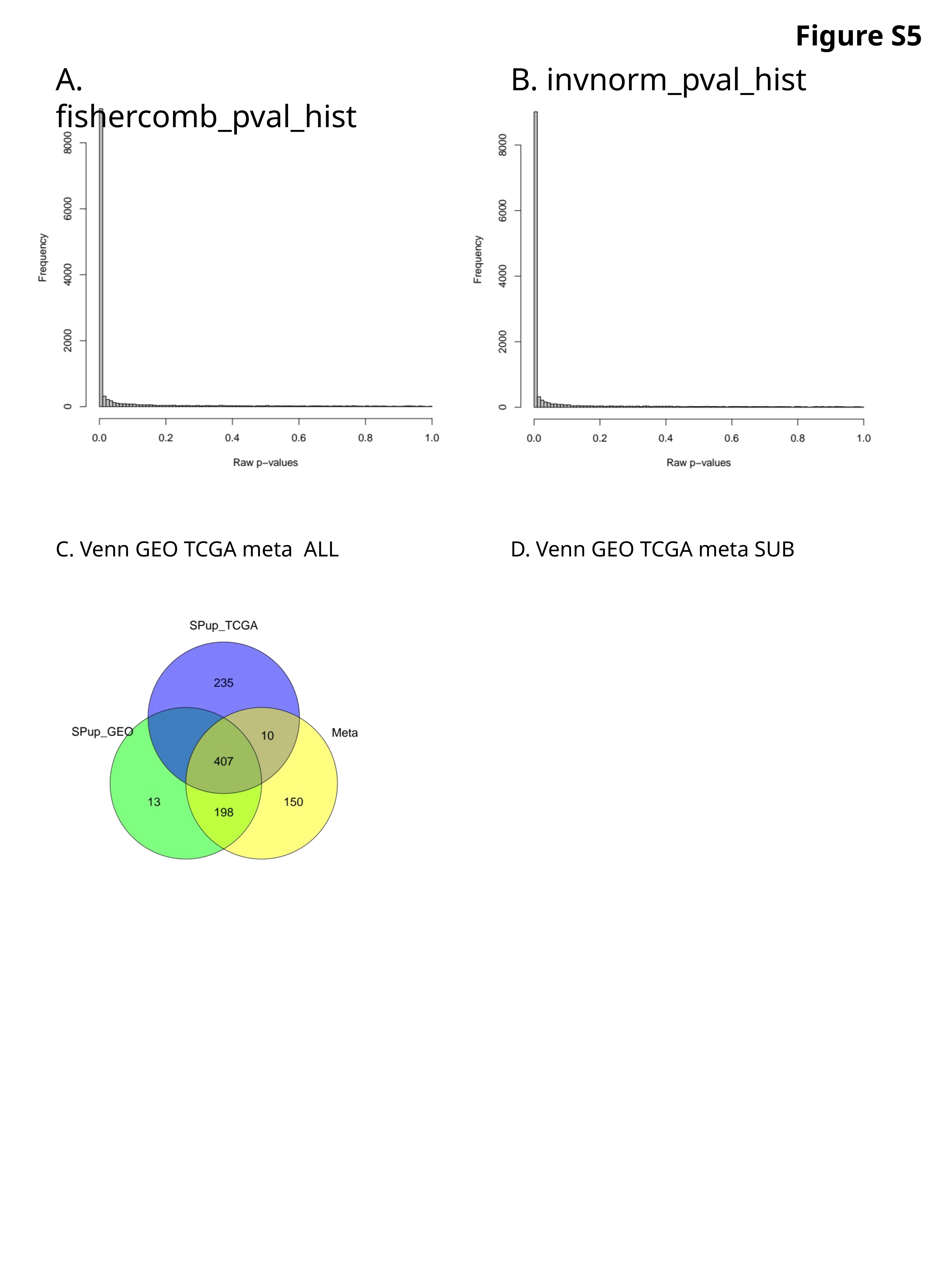

Figure S5
A. fishercomb_pval_hist
B. invnorm_pval_hist
C. Venn GEO TCGA meta ALL
D. Venn GEO TCGA meta SUB

## Slide 6
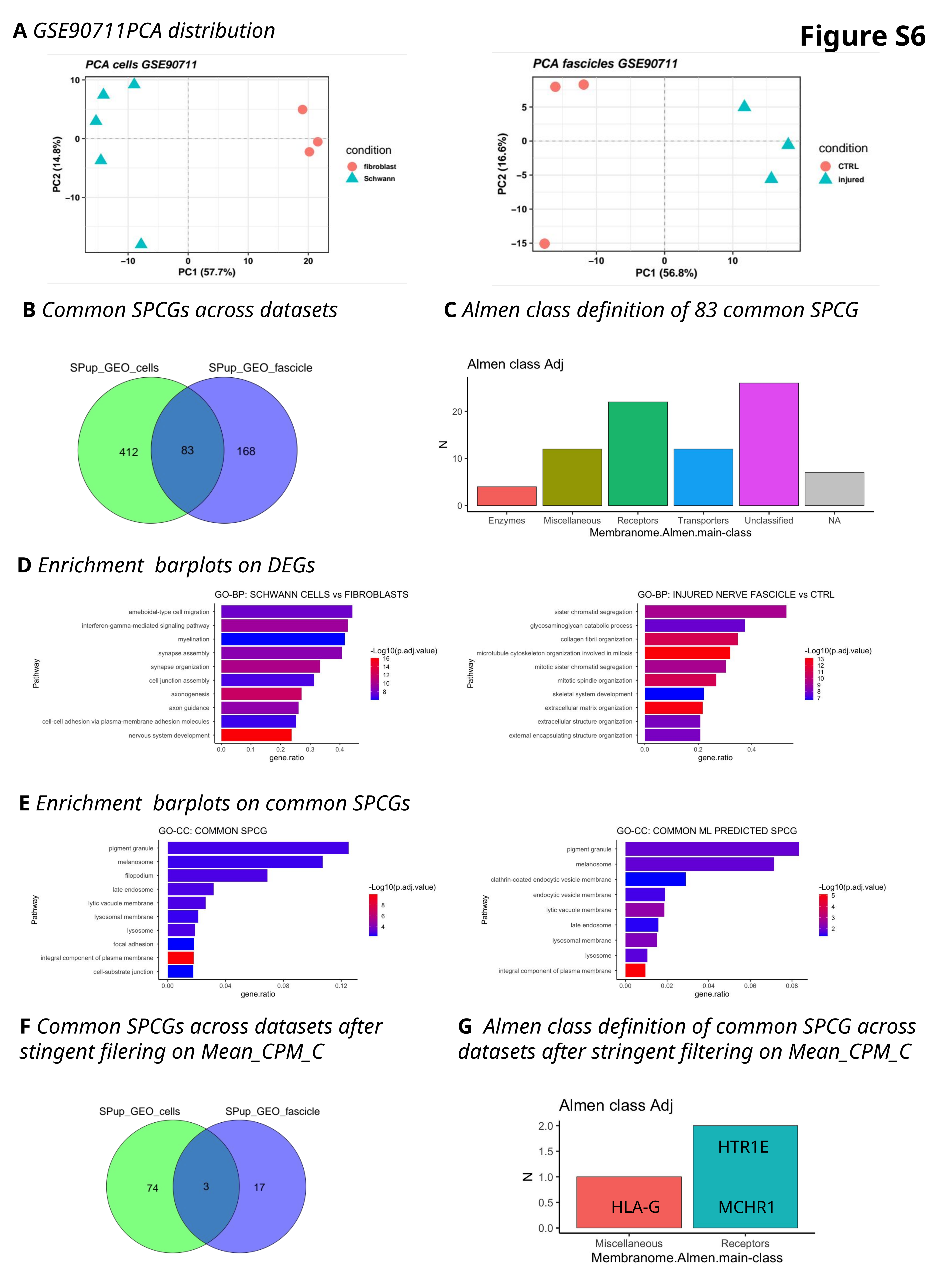

A GSE90711PCA distribution
Figure S6
B Common SPCGs across datasets
C Almen class definition of 83 common SPCG
D Enrichment barplots on DEGs
E Enrichment barplots on common SPCGs
F Common SPCGs across datasets after stingent filering on Mean_CPM_C
G Almen class definition of common SPCG across datasets after stringent filtering on Mean_CPM_C
HTR1E
HLA-G
MCHR1
